# Early-Life Exposure to Paraquat Aggravates Sex-Specific and Progressive Abnormal Non-Motor Neurobehavior in Aged Mice

**DOI:** 10.3390/toxics11100842

**Published:** 2023-10-07

**Authors:** Zhenzi Zuo, Jiayi Li, Bing Zhang, Ai Hang, Qiaoxu Wang, Guiya Xiong, Liming Tang, Zhijun Zhou, Xiuli Chang

**Affiliations:** 1 School of Public Health and Key Laboratory of Public Health Safety of the Ministry of Education, Shanghai Medical College of Fudan University, Fudan University, Room 233, Building 8, 130 Dongan Road, Shanghai 200032, China; xiaosanheizzz@sina.cn (Z.Z.); 21211020090@m.fudan.edu.cn (J.L.); 18211020115@m.fudan.edu.cn (B.Z.); neimenghangai2008@126.com (A.H.); gyxiong16@fudan.edu.cn (G.X.); zjzhou@fudan.edu.cn (Z.Z.); 2 Pharmacology and Toxicology Department, Shanghai Institute for Food and Drug Control, Shanghai 201203, China; xuxuwang8211@126.com (Q.W.); tangliming@smda.gov.cn (L.T.)

**Keywords:** paraquat, early-life, non-motor neurobehavior, gender differences, late life

## Abstract

Early-life exposure to environmental neurotoxicants is known to have lasting effects on organisms. In this study, we aim to investigate the impacts of PQ exposure during early developmental stages and adult re-challenge in aged mice on non-motor neurobehavior. Two mouse models, which were exposed once during early life stage and re-exposure at adulthood, were created to explore the long-term effects of PQ on non-motor neurobehavior. As the results showed, early-life exposure to PQ caused impairment in working memory and cognitive ability in aged male mice, but not in female mice, exhibiting a sex-specific impairment. Moreover, male mice that were re-challenged with PQ at adulthood following early-life exposure also exhibited non-motor neurobehavioral disorders. Notably, re-exposure to PQ exacerbated neurobehavioral disorders and anxiety levels compared to single exposure during different life stages. Collectively, early-life exposure to PQ can result in irreversible impairments in non-motor neurobehavior and increase susceptibility to subsequent insults in male mice, but not in female mice, suggesting greater sensitivity in male rodents to PQ-induced non-motor neurobehavioral deficits.

## 1. Introduction

Paraquat (1, 1-dimethyl-4, 4-bipyridium dichloride, PQ) is a broad-spectrum herbicide that is widely used in agricultural production with a low cost, high efficiency and non-selectivity [1,2]. However, the high solubility of PQ in soil poses a significant risk of soil and water contamination [3,4]. Even though it has been banned in more than 67 counties with reasons related to health and environmental hazards, high toxicity, and a lack of antidote that is frequently mentioned, PQ is still widely used in many other regions, particularly in Latin America and Asia [5]. Notably, the structure of PQ is similar to that of MPP+, the toxic metabolite of 1-methyl-4-phenylpyridinium (MPTP) [6], which is a neurotoxicant. Numerous epidemiological studies have shown that the prolonged exposure to low doses of PQ through farming, food and drinking water significantly increased the risk of Parkinson’s disease (PD), Alzheimer’s disease (AD) and other chronic neurodegenerative diseases [7,8,9]. Animal models have revealed that PQ can penetrate the blood–brain barrier of rodents, with the half-life lasting for 28 days [1], and can accumulate in various brain regions, including the hippocampus and cerebellum, potentially leading to selective attacks on dopamine neurons and PD-like symptoms [10]. Its neurotoxicity may be related to oxygen stress, mitochondrial abnormalities and energy dysfunction [11].

Parkinson’s disease (PD) is an age-related chronic progressive neurodegenerative disease, with the prominent feature of selective deterioration of dopaminergic neurons in the substantia nigra pars compacta (SNpc) of the midbrain [12,13], resulting in a reduction in dopamine (DA) levels in the striatum, which plays a key role in regulating the physiological functions of the central nervous system. Currently, PD is increasingly recognized as a systemic disorder [14], with the primary symptoms being static tremor, stiffness, slow movement and postural instability, among which non-motor deficits form an important part of the syndrome, including depression, anxiety, autonomic and cognitive impairment occurring in PD patients [13,14].

Although PD is typically considered an adult-onset disease, the possibility that it may be related to insults that occur earlier in life has been raised. Exposure to environmental contaminants during critical periods of neurodevelopment may induce profound influence and predispose individuals to developing PD [15]. Notably, the immature blood–brain barrier during early life stages, such as the embryonic, fetal and neonatal stages, leave the nervous system more susceptible to toxic effects of environmental neurotoxins [16]. Studies on animal models have provided compelling evidence that a range of environmental factors (pesticides like PQ or maneb (MB)) during the perinatal [17] and prenatal periods [18] can either directly cause a reduction in dopamine neurons, or increase susceptibility to degeneration with subsequent environmental insults, suggesting a potential relevance of PD on the fetal basis of adult disease. Several rodent studies have shown that PQ exposure during the postnatal period can reduce locomotor activity, rendering it more vulnerable to subsequent adult re-challenge with the same pesticides [17,19]. However, few studies have explored the impacts of early-life PQ exposure on non-motor neurobehavior in later life stages.

Gender is a significant risk factor for Parkinson’s disease, which is attributed to hormonal factors. Estrogens have been proven to enhance synaptic plasticity, reduce β-amyloid and counteract the expression of pro-apoptotic factors, owning to the properties of antioxidant benefits and neuroprotective function [20]. As studies have shown, men are twice as likely to develop Parkinson’s disease [21], with 1.5 times the prevalence [22] compared to women. In addition, male PD patients generally experience an earlier onset age, faster disease progression [14,23] and more severe dopaminergic demineralization [24]. These gender differences have also been observed in animal models of PD. Several studies have revealed that female rodents are less susceptible than males to the behavioral and neurodegenerative effects of DA-targeting toxins, such as MTPT and 6-hydroxydopamine (6-OHDA) [25,26,27]. Collectively, numerous findings have shown that the protection against PQ in females is conferred post-puberty, suggesting that maturation of reproductive systems may play a role in this response. However, limited research has focused on the effects of early-life exposure to PQ on non-motor behavior in animals of different genders.

Herein, we conduct animal experiments to investigate the impacts of early-life PQ exposure on non-motor neurobehavioral outcomes in male and female mice. Further, we focus on the differences of age and re-challenge after PQ exposure to establish a direct causal relationship between PQ exposure and impairments of non-motor neurobehavior in animal models. Our findings provide experimental evidence for assessing potential risks of PQ exposure at different life stages in humans.

## 2. Materials and Methods

### 2.1. Animal Model and Chemicals

Pregnant C57BL/6 mice were purchased from Shanghai Institute for Food and Drug Control, and mice were provided with diet and water ad libitum. Animals were maintained in standard conditions (12 h light/dark cycle) at a constant temperature (18–22 °C) and humidity (40–50%). All animal care and experimental procedures were conducted in accordance with Fudan University ethical guidelines. PQ was provided by Sigma-Aldrich (Milan, Italy), diluted with saline. Five days after birth, the mice were randomly divided.

### 2.2. Experimental Design and Exposure Protocols

#### 2.2.1. PQ Exposure in Early Life of Male and Female Mice

**Figure 1:**
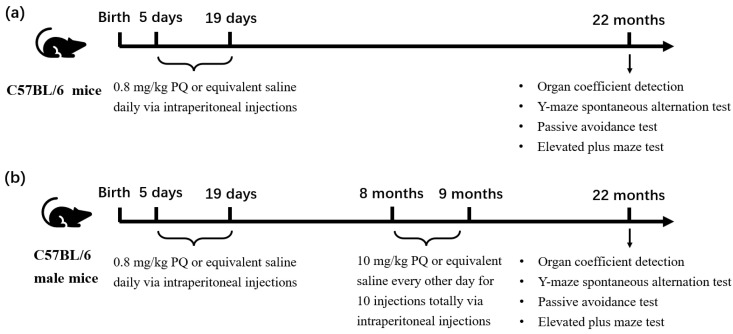
The protocols for animal treatment and processing that were employed in this study. The experimental schedules of early-life exposure model (**a**) and re-challenge model (**b**) are exhibited, respectively.

Both 5-day-old male and female pups were divided into two groups: NS group (*n* = 11–15 female mice/group, *n* = 9–18 male mice/group) or PQ group (*n* = 14–16 female mice/group, *n* = 8–23 male mice/group). Mice from NS group received intraperitoneal (i.p.) treatment of saline, and mice from PQ group were exposed to 0.8 mg/kg PQ every day (i.p.); the exposure time was 15 days. The dose (0.8 mg/kg) and duration of PQ exposure (15 days) were determined based on previous research on early-life PQ exposure in mice [28], together with our pretest. At 22 months old, all animals from the two groups were subjected to behavioral tests, including organ coefficient detection, y-maze spontaneous alternation, passive avoidance test and elevated plus maze test (Figure 1a).

#### 2.2.2. PQ Re-Exposure at Adulthood in Male Mice

Male mice were randomly divided into four groups, and exposed to either saline or PQ during early PN period (PN day 5–19) or in adulthood (8 months), respectively. The experimental schedule comprised four groups as follows:

a. NS + NS group (treated with saline at both PN period and adulthood, *n* = 9–18);
b. NS + PQ group (treated with saline at PN period and with PQ at adulthood, *n* = 8–15);
c. PQ + NS group (treated with PQ at PN period and with saline at adulthood, *n* = 8–23);
d. PQ + PQ group (treated with PQ at both PN period and adulthood, *n* = 15–18).

During the PN period, animals were administered PQ (0.8 mg/kg) or equivalent saline via i.p. injections on their fifth day of life and continued for fifteen days. During adulthood, animals were exposed to PQ (10 mg/kg) or equivalent saline via i.p. injections at 8 months of age every other day for a total of 10 injections. This PQ exposure concentration (10 mg/kg) is reported to accelerate the loss of midbrain dopaminergic neurons [1]. At 22 months of age, each group underwent behavioral tests, including organ coefficient detection, y-maze spontaneous alternation, passive avoidance test and elevated plus maze test. This experimental schedule was performed to investigate whether early PN exposure to the PQ renders neurobehavior of mice more susceptible to a re-challenge at adulthood (Figure 1b).

### 2.3. Y-Maze Spontaneous Alternation Test

The Y-maze spontaneous alternation test was used to evaluate spatial working memory in aged mice [29]. The Y-maze consisted of three arms (35 cm long, 5 cm wide and 15 cm high) and converged on an equilateral triangular at the central area. Mouse were placed individually in the center of the maze at random and were allowed to explore freely for 5 min. Activity of each mouse was tracked and recorded with the manufacture’s software. The maze was thoroughly cleaned between sessions. Consecutive entries into all three arms were considered as an alternation. The maximum spontaneous alternation was calculated by subtracting 2 from the total number of arms entered. The percent spontaneous alternation was defined as (actual alternations/maximum alternations) × 100%.

### 2.4. Passive Avoidance Test

The passive avoidance test was used to assess the learning and memory of aged mice [30]. We conducted passive avoidance test using a shuttle box, which is composed of two distinct chambers with a grid floor, one dark and one illuminated, separated by a guillotine door. The trial involved two separate trials. During the initial training trial, each mouse was located in the illuminated room and after 10 s, the guillotine door was opened. As soon as the mice entered the dark compartment, the door was closed immediately, and an electric foot shock (0.5 mA for 5 s) was delivered through the grid floor. Then, the door opened, allowing for the mice to escape into the light chamber. A total of 24 h after the acquisition trial, the mice were placed in the illuminated compartment again as training, and the latency to enter the dark chamber was recorded. The latency in both acquisition and retention trials was measured, with a maximum of 300 s.

### 2.5. Elevated plus Maze Test

The elevated plus maze test was utilized to evaluate the impact of PQ on the anxiety of aged mice [29]. The apparatus consisted of four arms with two open arms (25 × 5 cm) and two closed arms (25 × 5 cm), all symmetrical to the central platform (5 × 5 cm), elevated approximately 30 cm off the floor surface. Each mouse was placed on the center platform facing an open arm individually, and was allowed to freely explore the maze for 5 min. Each of the animal’s exploration was traced and recorded using the manufacture’s software. Several variables, including the total number of entries, time spent in open and closed arms, were analyzed.

### 2.6. Mice Organ Coefficient Detection

After completing behavioral tests, the mice were weighted and then euthanized for organ dissection. The brain, heart, liver, kidney and lungs were excised and weighed after, excluding the adipose tissue, to calculate organ coefficients. Organ coefficients were calculated as the ratio of organ wet weight to body weight.

### 2.7. Statistical Analysis

All data from this study were analyzed using one-way ANOVA. Following significant ANOVA, for further comparison, multiple post hoc comparisons were carried out using Least Significant Difference (LSD) test (*p* < 0.05). The results were reported as mean ± standard error of the mean (S.E.M), with *p* < 0.05 denoting statistical significance. Analysis was performed using IBM SPSS Statistics 22, and all graphics were created using the GraphPad Prism 8.0.

## 3. Results

### 3.1. Exposure to PQ Has No Impact on Body Weight and Organ Coefficients in Aged Mice

To investigate the systemic toxic effects of PQ in mice, body weight and organ coefficients were detected in our two models. No significant differences were observed in body weight and organ coefficients of brain, heart, liver, kidney and lung between NS and PQ groups, in either male or female mice (Figure S1), suggesting that the dose of 0.8 mg/kg PQ exposure did not result in systemic toxicity. In the re-challenge model of mice, no significant differences were observed in body weight and organ coefficients of the brain, heart, kidney and lung, despite some fluctuations in liver coefficients (Figure S1). Overall, exposure to PQ in this study did not result in systemic toxicity in C57BL/6 mice.

### 3.2. Exposure to PQ during Early Life Elicits Sex-Specific Abnormal Non-Motor Neurobehavior in Aged Mice

To investigate gender differences in early-life exposure to PQ, we conducted a batch of behavioral experiments on aged male and female mice, following 15 consecutive days of exposure to 0.8 mg/kg PQ during the PN period.

**Figure 2:**
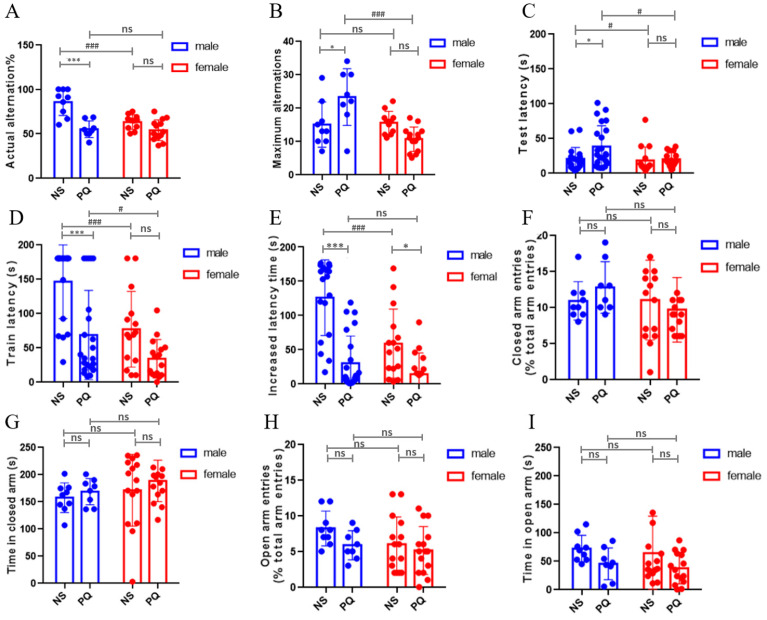
Early-life PQ exposure induced gender-specific abnormal non-motor behavior in aged mice among NS and PQ groups. In Y-maze test (*n* = 8–14 mice/group, **A**,**B**), locomotor activity was measured by collecting the ratio of actual alternation (**A**) and the number of maximum alternations (**B**), which determine the spatial working memory of mice. In passive avoidance tests (*n* = 15–23 mice/group, **C**–**E**), the test latency (**C**), train latency (**D**) and increased latency (**E**) were observed to evaluate the cognitive behavior of mice. In terms of elevated plus maze test (*n* = 8–15 mice/group, **F**–**I**), closed arm entries (**F**), open arm entries (**H**) and the time spent in open (**I**) or closed arm (**G**) were recorded, respectively, to assess the anxiety levels of mice. Data are presented as mean ± SEM. * *p* < 0.05, *** *p* < 0.001 compared between treatment groups. ^#^ *p* < 0.05, ^###^ *p* < 0.001 compared between sexual groups. ns, no statistically significant (*p* > 0.05).

The Y-maze test was carried out to determine the impact of PQ on the development of spatial working memory. Significant statistical differences were observed in actual alternation ratios (*p* < 0.001, Figure 2A), and maximum alternations (*p* < 0.05, Figure 2B) in male mice between NS and PQ groups, exhibiting a reduction in actual alternation and an increase in maximum alternations; however, these differences were not observed in female mice (Figure 2A,B). In the PQ exposure groups, the maximum alternations in male mice were significantly increased compared to female mice (*p* < 0.001, Figure 2B). These data indicate that exposure to 0.8 mg/kg PQ at early life stage (PN day 5–19) impacts short-term memory in late-life male mice, but not in female mice.

The passive avoidance tests were administered to evaluate the impact of PQ on cognitive behavior. As the results show, significant differences were observed in male mice between NS and PQ groups (*p* < 0.05, Figure 2C; *p* < 0.001, Figure 2D,E). The test latency and train latency were comparable between female mice in NS and PQ groups (Figure 2C,D), with significant differences observed in the increased latency time (*p* < 0.05, Figure 2E). In the exposure groups, the test latency and train latency in male mice were decreased compared to female mice (*p* < 0.05). Overall, exposure to PQ during early life stage had less effect on the cognitive behavior of aged female mice.

The elevated plus maze test was conducted to examine the influence of PQ exposure on the anxiety of mice. However, we did not find any significant differences among all the groups (Figure 2F–I). Both NS and PQ groups, and male and female groups, had similar open/closed arm entries (Figure 2F,H) as well as similar times spent in open and closed arm (Figure 2G,I), suggesting that PQ exposure during early life stage did not cause excessive anxiety in aged male and female mice.

### 3.3. Re-Exposure to PQ at Adulthood after PN Exposure Aggravated Spatial Working Memory Impairment in Aged Male Mice

**Figure 3:**
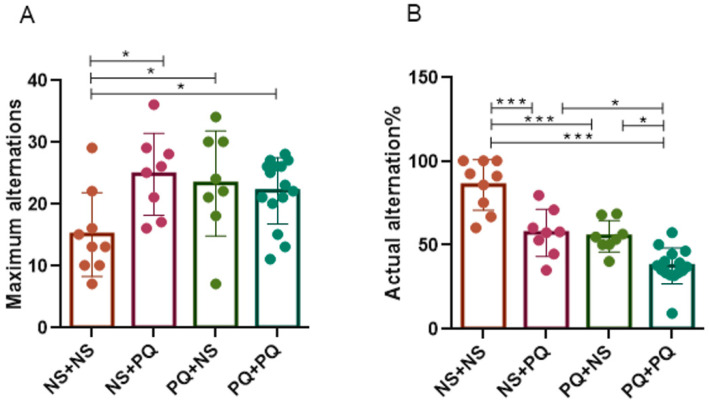
PQ exposure in different life stages impaired spontaneous alternation in aged male mice in the Y-maze test. The number of maximum alternations (**A**) and the ratio of actual alternation (**B**) in the maze were reported, which indicate the spatial working memory of mice. Data are presented as mean ± SEM. *n* = 7–15 mice/group. * *p* < 0.05; *** *p* < 0.001.

The Y-maze task is a widely used test to assess spatial recognition memory in rodents by allowing for continuous spontaneous alternation [31]. Significant statistical discrepancies were observed between NS + NS group and other exposure groups. To detail, NS + PQ, PQ + NS and PQ + PQ mice exhibited a decrease in maximum alternations (all *p* < 0.05, Figure 3A) and an increase in actual alternation ratios (all *p* < 0.001, Figure 3B) when compared to NS + NS group, suggesting the impairment of spatial working memory induced by PQ exposure whether in early life stage or adulthood. Remarkably, the PQ + PQ group showed a reduction compared to NS + PQ and PQ + NS groups (*p* < 0.05, Figure 3B), which emphasized an increased risk of re-exposure to PQ on neurobehavior disorder. These results suggest that PQ exposure at different stages of life impairs spatial working memory in late-life male mice, with a greater impact seen in those exposed to PQ repeatedly during both PN period (0.8 mg/kg PQ, PN days 5–19) and adulthood (10 mg/kg PQ, 8 months).

### 3.4. Re-Exposure to PQ at Adulthood after PN Exposure Results in Progressive Cognitive Impairments in Aged Male Mice

**Figure 4:**
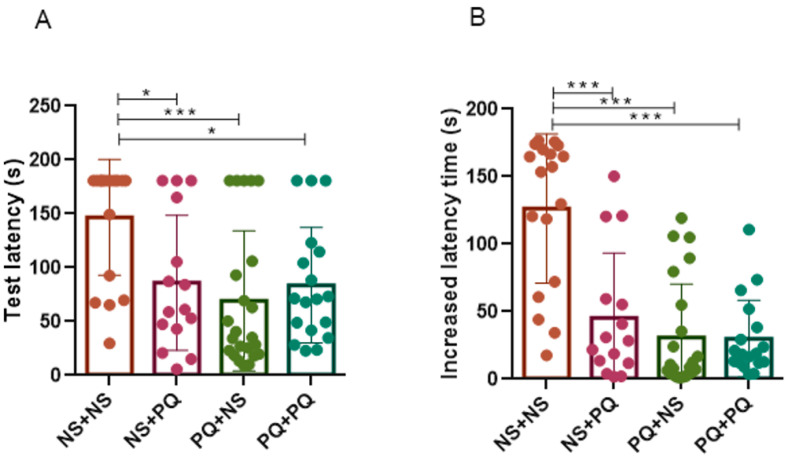
PQ exposure at different life stages induced cognitive deficit in aged male mice in the passive avoidance tests. Test latency (**A**) and increased latency time (**B**) were detected in this experiment, suggesting short-term cognition and memory. Data are presented as mean ± SEM. *n* = 15–23 mice/group. * *p* < 0.05; *** *p* < 0.001.

The passive avoidance task was considered a representative approach to evaluating cognitive behavior in mice (such as short-term reference memory), with a well-established experimental procedure [32]. Here, we performed a passive avoidance task on mice to assess the impact of PQ re-exposure on short-term cognition and memory. The cognitive ability was evaluated through latency, which involves both training and testing at 24 h intervals. In this way, we observed notable differences between PQ-treated groups and control group. Overall, compared to NS + NS group, the latency period was significantly reduced in NS + PQ (*p* < 0.05), PQ + NS (*p* < 0.001) and PQ + PQ (*p* < 0.05) groups (Figure 4A). Still, no significant differences were noticed between the groups with different PQ treatments. Additionally, an increased latency time was shown to be statistically different between NS + NS group and NS + PQ, PQ + NS, PQ + PQ groups (all *p* < 0.001, Figure 4B). Although there was a decreasing trend in the three PQ-treated groups, the differences were not statistically significant. These findings indicate that male mice treated with PQ at different stages of life (0.8 mg/kg PQ at PN days 5–19 or 10 mg/kg PQ at 8 months) exhibited deficiencies at a late life stage in passive avoidance tasks.

### 3.5. Re-Exposure to PQ at Adulthood after PN Exposure Exacerbated Anxiety in Aged Mice

**Figure 5:**
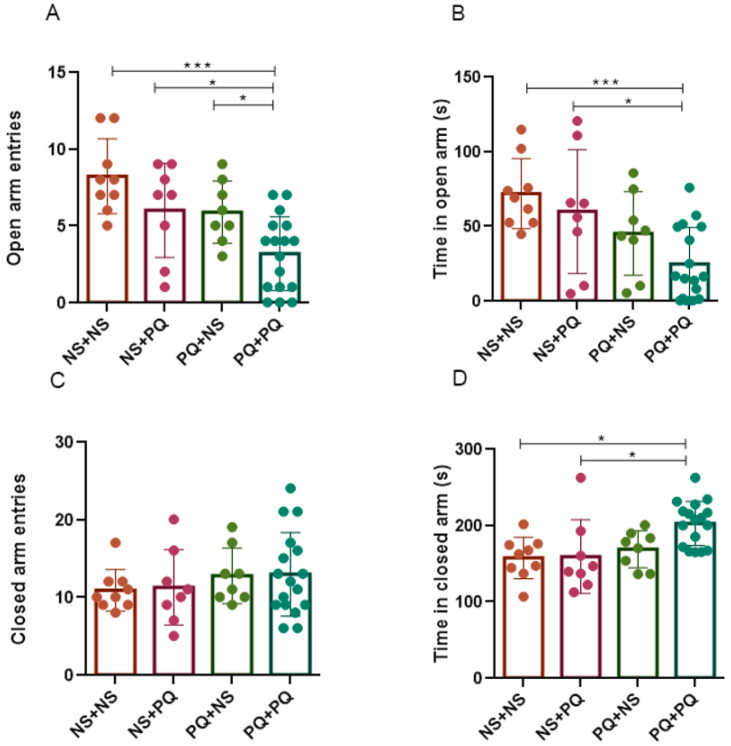
PQ exposure at different life stages induced anxiety in aged male mice in the elevated plus maze tests. The number of entries into the open arm (**A**) or closed arm (**C**) and the time spent in the open arm (**B**) or closed arm (**D**) were reported in this experiment, which indicated the anxiety level of mice. Results are presented as mean ± SEM. *n* = 8–17 mice/group. * *p* < 0.05; *** *p* < 0.001.

To evaluate the impact of PQ exposure and re-exposure on emotional response, we conducted the elevated plus maze test [33] to explore the anxiety level of mice, which is determined by the number of entries and the time spent in open or closed arms. As shown, PQ + PQ group exhibited a significant reduction in the number of open arm entries and time spent in the open arm (Figure 5A,B), and an increase in the time spent in the closed arm (*p* < 0.05, Figure 5D), compared to NS + NS group and NS + PQ group, suggesting an elevated anxiety level in mice who were re-exposed to PQ at adulthood after PN period exposure. Moreover, although PQ + NS group exhibited a decrease in the number of open arm entries compared to PQ + PQ group (*p* < 0.05, Figure 5A), no statistical differences were observed in other parameters (Figure 5B–D). Thus, we concluded that a single exposure to PQ during early life stage does not affect the emotional adaptability of adult mice, but increases their susceptibility to anxiety upon re-exposure to PQ during adulthood. In general, re-exposure to PQ at adult stages (10 mg/kg PQ at 8 months) after PN period exposure (0.8 mg/kg PQ at PN days 5–19) exacerbated anxiety levels in late-life male mice.

## 4. Discussion

In this study, two models of PQ exposure in mice are presented here. In the first model, male and female pups were exposed to normal saline and PQ (0.8 mg/kg) during the PN period. The results reveal sex-specific differences: male mice exposed to PQ exhibited abnormal non-motor neurobehavior, while female mice did not. To further investigate the effects of early-life PQ exposure on non-motor neurobehavior in a late life stage, the second model (re-challenge model), in which male mice were exposed to saline or PQ during early life (PN days 5–19) or adulthood (8 months), respectively, was established. The data show that male mice exposed to PQ resulted in the impairment of cognitive behavior and spatial working memory whether during PN period or adulthood. Remarkedly, greater impairments were observed on these non-motor neurobehaviors in mice’s re-exposure to PQ. In addition, anxiety levels were significantly increased only in mice that were re-exposed to PQ. Collectively, these results suggest that PQ exposure at early life stage can produce progressive and irreversible non-motor neurobehavioral impairments, and enhance susceptibility to subsequent PQ insults.

PQ is a non-selective herbicide that can contaminate the environment, leading to human exposure through ingestion of contaminated food and water, inhalation and dermal contact [34,35]. Taking into account the high levels of circulating PQ (~4.8 μg/mL) linked to survival in cases of human poisoning [36], as well as the oral LD_50_ of 150 mg/kg body weight in rats, together with the pretest, we administered a low dose of PQ (0.8 mg/kg) to young male and female mice aged PN 5–19. To further investigate the effects of re-challenge during adulthood, a relatively high dose of 10 mg/kg of PQ was chosen, and this particular concentration was reported to promote the loss of midbrain dopaminergic neurons [1], which is reminiscent of what occurs in PD. Based on the results of body weight and organ coefficients, we can conclude that the concentration of 0.8 mg/kg PQ in early life stage did not produce systemic toxicity.

It is now widely acknowledged that PD is characterized not only by motor symptoms, but also by various non-motor symptoms, including anxiety and depression. Non-motor symptoms have a greater impact on health-related quality of life than motor abnormalities [37,38]. Furthermore, PD is a neurodegenerative condition that typically onsets in late life stage, with morbidity potentially linked to earlier life insults. In daily life, diagnosing PD can be challenging, and determining the span of exposure is difficult [39]. In this study, we administered PQ at different life stages in mice, particularly during early life stage, and observed the effect on aged male and female C57BL/6 mice. During the development period, PQ exposure greatly influenced cognition and working memory in male mice, but not in female mice, which aligns with prior studies that reported that gender differences play a role in PQ tolerance. In detail, the genetic architecture for PQ susceptibility in Drosophila appears to be related to gender differences, with more than 90% of the associated genes being sex-specific [40]. In *T. californicus*, males and females exhibited different transcriptional profiles to PQ, where males expressed over four times as many genes in response to exposure, including heat shock proteins, antioxidant genes and protease genes [41]. Additionally, previous evidence suggests that female gonadal steroids, particularly estrogens, possess potent anti-inflammatory [42,43] and neuroprotective effects, and also reduce the cortical infarction volume in stroke rodent models [44]. In addition, estrogens were able to effectively reduce the risk of developing AD or delay onset in a population study [20]. To rule out the influence of these hormones, we chose to expose rodents to PQ during early postnatal stage, before the reproductive system fully develops. Nevertheless, our data still reveal that male mice were more vulnerable than females to the non-motor neurobehavioral dysfunction induced by PQ, suggesting that there may be additional underlying mechanisms that play roles in the gender differences in neurobehavioral abnormalities induced by PQ beyond hormonal protective factors, which is consistent with the findings of Quinn et al. [45], who discovered that whether the gonads are present or not, female mice (sex chromosome XX) showed faster food reinforced instrumental habit formation than male mice (sex chromosome XY), and more research needs to be carried out on the underlying mechanism. Furthermore, dysregulation of the nigrostriatal DA system induces non-motor neurobehavior and emotional changes, such as anxiety and depression. Depression and anxiety are prevalent behavioral symptoms of PD, with an estimated prevalence of 40%, and both symptoms typically coexist [38,46]. However, research investigating the non-motor neurobehaviors of PD remains limited. Therefore, pesticide-based PD models might constitute a more reliable system to examine various aspects of PD [47].

A number of studies have demonstrated that neurodegeneration is associated with senescence and neurogenic dysfunction caused by neurotoxic agents [48]. PQ can generate large amounts of reactive oxygen species (ROS) and selectively eradicate dopaminergic neurons in the substantia nigra [3,49]. Interestingly, a significant loss of dopaminergic cells appears to require repeated exposures within a relatively short time period—which is consistent with a “two-hit” hypothesis. Based on the “two-hit” hypothesis, the initial exposure may sensitize the system to subsequent exposure by activating microglia cells [50]. The neurotoxicity caused by PQ exposure in early life stage can be counteracted by the repair of the nervous system. However, it may also enhance susceptibility to neurotoxicants in later life stage, leading to the occurrence of neurodegenerative diseases. The animals were more susceptible to neuronal damage when re-exposed to PQ at adulthood, accompanied by impaired motor function [17]. This phenomenon indicates the occurrence of “silent neurotoxicity”, which was firstly introduced by Reuhl [51], as a proposed mechanism for adult-onset disease with potential developmental origins. This concept was hypothesized to be responsible for increases in adult susceptibility to environmental factors, and was proven to be associated with the occurrence of neurodegenerative diseases [52,53]. A multiple-hit hypothesis concerning the impact of multiple risk factors on the brain may be further considered, as it could also play a fundamental role in neurotoxicity [54,55]. The theory postulates that insults to different target sites within a particular brain system, whether occurring concurrently or cumulatively, can compromise homeostatic and repair capacities of the system, and thereby increase its vulnerability. Similarly, our findings show that developmental exposure to PQ can render neurotoxicity, such as cognition and working memory impairments, and heighten susceptibility to a following adult re-challenge with the same toxicant, while the specific mechanism remains to be further studied and clarified. These findings provide new insights for assessing cumulative toxicity and repeated insults.

The developmental period is a critical phase for central nervous system maturation, where individuals are highly susceptible to environmental factors, which have been implicated in the pathogenesis of PD, AD or other neurodegeneration diseases [17,56,57]. Studies have shown that PQ is capable of penetrating the blood–brain barrier of rodents and damage nigrostriatal dopaminergic neurons [3,8], which are crucial for maintaining normal non-motor neurobehavior. The underlying mechanism may be related to the transportation of microglia, which can uptake PQ into dopaminergic neurons. This process involves the reduction of PQ^2+^ to PQ^+^ by enzymes (like NOX on microglia) so as to enter the dopamine transporter (DAT) [3,58]. Once crossing the blood–brain barrier, PQ can enter the neutral amino acid transport system and be transferred to nerve cells in the form of Na^+^ dependence and persist in the mouse brain, with an apparent half-life of approximately 4 weeks [1]. Widdowson reported that the concentration of PQ in the brains of newborn mice was significantly higher than that of adult and elderly mice when administered with PQ at different life stages simultaneously [59], suggesting that PQ impairs the integrity of the blood–brain barrier in newborn mice. To further investigate the potential impact of developmental insults on Parkinson-like symptoms, we utilized male mice to establish another PQ exposure model for the following experiments: Male mice were divided randomly into four groups, in which they were exposed to PQ during different life stages, and the behavioral outcomes were investigated. As PD typically manifests late in life, we raised mice to old age to explore the non-motor neurobehavior. Compared to saline-treated group, three PQ-treated groups showed impairments in cognitive behavior and working memory. Notably, mice that were re-exposed to PQ during adulthood following exposure during the PN period displayed severe anxiety and deficits in working memory. Despite the absence of statistically significant differences, a changing trend was observed between PQ + NS and NS + PQ groups. Recent studies have reported that exposure to PQ during the critical period of rapid brain development such as fetal or neonatal periods may lead to structural and functional abnormalities of the central nervous system and impact neurological behavior in adulthood, and further increase the risk of developing PD later in life [15,17], which is in accordance with our findings. All these data suggest that early-life exposure to PQ can lead to progressive and permanent neurotoxicity and increases susceptibility to subsequent re-exposure in adulthood with the same toxicant.

Since significant results have yet to be observed in the effects of single pesticide exposure, combined exposure models were adopted in many experiments. In a study conducted by Dirleise Colle, male Swiss mice were exposed to a combination of 0.3 mg/kg PQ and 1.0 mg/kg MB from postnatal days 5 to 19, and a parallel group was re-challenged at 3 months of age with 10 mg/kg PQ and 30 mg/kg MB. Their motor function was evaluated at the postnatal day 138, revealing significant deleterious consequences from early-life exposure that persisted until adulthood [17]. In another developmental model of pesticide exposure [18], pregnant mice were exposed to MB (1.0 mg/kg) during gestational days 10–17, which increased their vulnerability to a re-challenge with PQ (5.0 mg/kg) at the age of 7–8 weeks. Although most experiments were conducted with youth or middle-aged mice, we aged the mice to simulate a PD model since PD typically onsets in a late life stage. Our findings suggest that insults occurring early in development can have long-term and delayed consequences for the DA system [17], highlighting the importance of including childhood pesticide exposure in epidemiologic studies and further evaluation in experimental models.

Although studies have indicated that PQ may play a role in the pathogenesis of PD, there is currently no research on the relationship between PQ exposure and non-motor neurobehavior, one of the typical manifestations of PD. This is the first study that focuses on the impairment of non-motor neurobehavior induced by PQ exposed at different life stages in mice using two exposure models. In this study, early-life exposure to PQ resulted in non-motor neurobehavior symptoms in late-life male mice, but not in female mice, exhibiting a sex-specific impairment. Notably, re-challenge with PQ at adulthood following early-life exposure exacerbated non-motor neurobehavior disorders and anxiety in aged male mice. Our study provides evidence for assessing the potential risks of PQ exposure at different life stages, especially at childhood, as well as re-exposure at adulthood.

Several limitations need to be mentioned in this study. Firstly, the exposure conditions are relatively restricted. While we made an effort to simulate potential exposure scenarios, it was difficult to recreate the multi-, complex exposure environments that humans encounter, whether in terms of the variety of chemical exposures or the exposure doses of PQ. Secondly, we merely focused on the neurobehavioral effects on individuals; however, multiple perspectives at the molecular or cellular level need to be explored to gain insight into the underlying mechanisms. Last but not least, the phenomena we observed in rodents cannot be extrapolated directly to humans due to species differences.

## 5. Conclusions

This study demonstrated that PQ exposure induced sex-specific and progressive abnormal non-motor neurobehavior in aged C57BL/6 mice, among whom young mice were found to be more susceptible to PQ exposure than adults. Furthermore, early-life exposure to PQ increased vulnerability to subsequent insults in male mice compared to female mice—this finding was obtained from the results of the Y-maze and passive avoidance tests.

## Data Availability

No new data were created or analyzed in this study. Data sharing is not applicable to this article.

## Supplementary Materials

The following supporting information can be downloaded at https://www.mdpi.com/article/10.3390/toxics11100842/s1, Figure S1: Effects of PQ exposure on body weight and organ coefficients in mice.
