# Supplementary material for: Early-Life Exposure to Paraquat Aggravates Sex-Specific and Progressive Abnormal Non-Motor Neurobehavior in Aged Mice
Source: Toxics. 2023 Oct 7;11(10):842. doi: 10.3390/toxics11100842 (PMC10611227; doi:10.3390/toxics11100842)
Supplement: Supplementary file 1 [file toxics-11-00842-s001.zip › toxics-2614429-SI.pdf]

toxics

Supplementary material: Early Life Exposure to Paraquat Ag-gravates Sex-specific and Progressive Abnormal Non-motor Neurobehavior in Aged Mice

Zhenzi Zuo, Jiayi Li, Bing Zhang, Ai Hang, Qiaoxu Wang, Guiya Xiong, Liming Tang, Zhijun Zhou and Xiuli Chang

1. Body Weight and Organ Coefficients

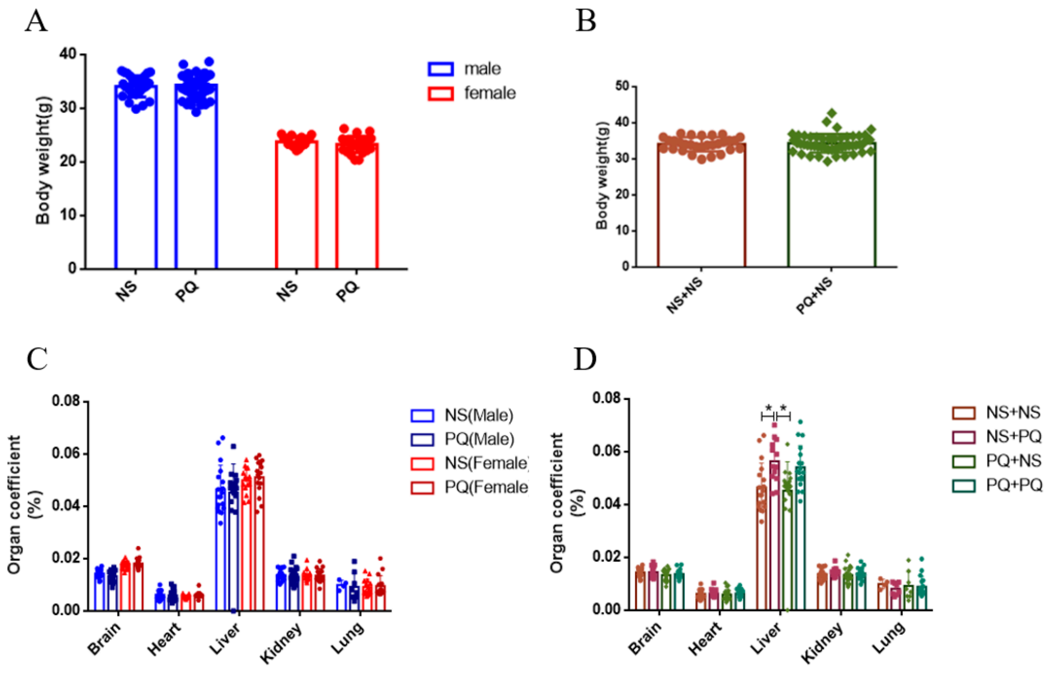

Figure S1. Effects of PQ exposure on body weight and organ coefficients in mice. The body weight (**A**) and organ coefficients (**C**) of brain, heart, liver, kidney and lung in male and female mice after exposure to 0.8 mg/kg PQ during PN period are presented. Similarly, the bodyweight of NS+NS and PQ+NS groups (**B**), and the organ coefficients (**D**) of brain, heart, liver, kidney and lung in male mice from re-exposure model that exposed to PQ or saline during different stages of life are shown. n=13-50 mice/group. Data are presented as mean ± SEM. *$p < 0.05$.
